# Monthly Summary

**Published:** 1887-04

**Authors:** 


### Monthly Summary.

---

Resorcin Inoculations in Phlegmon.—Dr. Ludwig Weiss, of New York, *Medical Record*, recommends very highly resorcin inoculations in Phlegmon, especially of the fingers. He claims it is an effective remedy in all furuncular and phlegmonous inflammations; it abates the inflammation, if used in time, by destroying the germ locally, as well as in the lymph canals leading from the point of infection, at the same time acting as an anæsthetic on the sensitive terminal nerve filiments. Dr. Weiss makes a number of shallow, parallel incisions through the integument into and around the lesion, then applies a thick layer of 10 to 30 per cent. resorcin lanoline ointment, then a piece of lint smeared with the ointment, then gutta-percha tissue, absorbent cotton, and a moist gauze bandage. The resorcin ointment must be applied abundantly and continuously. The ointment must be renewed twice daily. Relief from pain and tension ensues in from six to twelve hours.

---

Sedative Cough Mixture.—When Dr. H. C. Wood recommends anything it is a guarantee of its merit. Hence we take the following from the *Therapeutic Gazette:*

Potassi citratis........................1 drachm.

Succi limonis...........................2 drachms.

Syr. ipecac.............................½ ounce.

Syr. simplicis..........................6 ounce.

A tablespoonful from 4 to 6 times a day.

When there is much cough or irritability of the bowels, paregoric may be added.

---

Warts.—The *Medical Press* says: "It is fairly established that the common wart, which is so unsightly and often so proliferous on the hands and face, can be easily removed by small doses of sulphate of magnesia taken internally. M. Colrat, of Lyons, has drawn attention to this extraordinary fact. Several children treated with three-grain doses of Epsom salts, morning and evening, were promptly cured. M. Aubert cites the case of a woman whose face was disfigured by these excrescences and who was cured in a month by a drachm and a half of magnesia taken daily. Another medical man reports a case of very large warts which disappeared in a fortnight from the daily administration of ten grains of salts."

The following formula, a modification of that recommended by M. Vigier for corns, is largely used by Vidal for warts:

| | | |
|---|---|---|
| Acid, salicylic | 1 | gramme. |
| Alcohol, 90° | 1 | " |
| Ether | 2½ | " |
| Collodion | 5 | " |

The solution should be painted over the affected surface each day.

---

GRIND IT OFF.—Should a devitalized tooth, after having been filled, become sore to the touch, rendering occlusion of the jaws painful, there is no better remedy than to grind away the tooth sufficiently to prevent occlusion. We have, in this way, several times prevented what promised to be severe periodontitis.—Dr. Catching, in *Southern Dental Journal.*

